# Abdominal Obesity, Testosterone Deficiency and Sedentary Lifestyle in Young Men from the General Population: The Relationship with Semen Quality, Hormonal and Metabolic Status

**DOI:** 10.3390/biomedicines14092097

**Published:** 2026-09-17

**Authors:** Ludmila Osadchuk, Alexander Osadchuk

**Affiliations:** Department of Human Molecular Genetics, Federal Research Center ‘Institute of Cytology and Genetics’, The Siberian Branch of the Russian Academy of Sciences, 630090 Novosibirsk, Russia; osadchuk@bionet.nsc.ru

**Keywords:** abdominal obesity, semen quality, sperm DNA fragmentation, reproductive hormones, metabolic indicators, testosterone deficiency, sedentary lifestyle, general population

## Abstract

**Background:** The demographic crisis observed in industrialized countries has been accompanied by a decline in the reproductive potential of human populations. One of the causes for the global deterioration in men’s reproductive health is adiposity and sedentary lifestyle, leading to reduced fertility and a decrease in androgen status. The aim of the study was to assess the association between abdominal obesity and semen parameters, including sperm DNA fragmentation, as well as key indicators of hormonal and metabolic status in young men from the general population. **Methods:** Our cross-sectional study was conducted among Russian and Belarusian men living in six cities. The examination of men (n = 1283, median age 23 years) included questionnaires, anthropometry, and the collection of peripheral blood and semen samples. Semen analysis was conducted according to WHO guidelines. Sperm DNA fragmentation index (DFI) was evaluated using the SCSA method. The serum and seminal zinc concentration was determined using spectrophotometry and direct calorimetry. Hormonal and metabolic levels were measured using commercial kits. Waist circumference (WC) was used as a surrogate marker of abdominal adiposity. Participants were allocated to three phenotypes: non-obese (control, WC < 94 cm), excess abdominal fat (94 ≤ WC < 102 cm) and abdominal obesity (WC ≥ 102 cm). **Results:** In our study population, 85.8% of participants were young (under 30 years of age), 8.6% had excess abdominal fat, 5.7% had abdominal obesity (AO), and 65.2% had normozoospermia. Men with AO were characterized by a decrease in semen volume, total sperm count, sperm concentration and progressive motility compared to the non-obese control. DFI was significantly higher in obese compared to non-obese men. Obese participants had significantly lower testosterone and inhibin B levels, but higher FSH and leptin levels compared to non-obese men. Men with AO had elevated serum zinc levels but reduced seminal zinc content. Abdominal obesity was also associated with significant metabolic changes, in particular, with increased levels of triglycerides, total cholesterol, low-density lipoproteins, fasting glucose and uric acid. AO-related testosterone deficiency (serum testosterone level ≤ 12.1 nmol/L) was detected in 38.7% of obese men and was accompanied by elevated DFI, levels of FSH, and zinc, as well as unfavorable changes in metabolic indicators. Physically active AO men (who engaged in recreational sports or physical labor) demonstrated reduced anthropometric indicators of AO and leptin levels, increased LH and testosterone levels, and improved carbohydrate and lipid metabolism compared to sedentary AO men. **Conclusions:** AO is accompanied by a decrease in the activity of Leydig and Sertoli cells, as well as an imbalance of the hypothalamic–pituitary–testicular axis and metabolic status. AO, associated with testosterone deficiency or a sedentary lifestyle, is a potential risk factor for the weakening of male reproductive health. However, physically active stout men showed more favorable anthropometric, hormonal, and metabolic indicators compared with sedentary stout men. In the long term, obese men who ignore a correction of sedentary lifestyle are not protected from a decline in reproductive health.

## 1. Introduction

In recent decades, epidemiological studies have accumulated significant evidence of a secular trend in male fertility. In various regions of the world, there have been observations of a negative trend in sperm quality and total testosterone levels, as well as an increase in the male factor in infertile couples, testicular cancer, and congenital abnormalities in the male reproductive system [1,2,3,4,5]. This universal decline in male fertility may be due to environment, modern lifestyle, and genetic factors that can potentially lead to irreversible reductions in male fertility. Despite the many causes and risk factors associated with reduced reproductive potential in men, there is a growing focus on the impact of lifestyle factors on male fertility [2,5].

One of the most frequently discussed reasons for the secular trend in men’s reproductive potential is obesity. According to a WHO report, in recent decades, there has been a significant increase in the number of people with overweight and obesity worldwide. In 2010, there were 1.6 billion (36% of the adult population) overweight and obese people (BMI over 25 kg/m^2^), while in 2015 there were about 2 billion (40%) such people. According to recent estimates, by 2030, almost 3 billion adults (about 50%) will suffer from overweight or obesity [6]. A similar trend was observed regarding the increase in the number of obese men (BMI ≥ 30 kg/m^2^): 9% in 2010, 11% in 2015, and 17% in 2030 [6]. The results of the “Global Burden of Disease” project also confirmed that the obesity rate has increased both at the global and regional levels in all countries [7]. In 2021, an estimated 1.00 billion adult males and 1.11 billion adult females had overweight and obesity. China had the largest population of adults with overweight and obesity, followed by India and the USA [7]. If obesity rates continue to rise, half of the world’s population (over 4 billion people) will be overweight or obese by 2035 [7].

Many researchers consider the long-term changes in men’s diet (consumption of high-calorie foods) and general physical activity (sedentary leisure pursuits), which contribute to abdominal fat accumulation, as significant causes of the negative trend in male reproductive potential observed over the past half-century [8,9,10,11]. The multifactorial nature of obesity involves interactions between the genome, environment, and lifestyle; however, while genetic factors contribute to obesity, they cannot explain the rapid increase in obesity in recent decades.

The WHO defines overweight and obesity as abnormal or excessive fat accumulation that presents a risk to health [12]. Obesity should be regarded as a chronic, progressive, and relapsing disease with multifactorial pathogenesis, including genetic, environmental, metabolic, and behavioral components. The obesity pandemic is accompanied by a rise in the number of complications and comorbidity: a total of 21 non-overlapping conditions have been identified [13]. Importantly, individuals with morbid obesity have a reduced life expectancy and an increased risk of all-cause mortality [14,15,16].

To date, a number of studies have been published on the adverse impact of obesity on various aspects of male reproductive function, contributing to a decline in male fertility and an increased risk of infertility. These include reduced semen quality and testosterone levels [7,9,17,18,19], the development of hypogonadism and erectile dysfunction [9,17,20,21,22,23], a disruption of the hypothalamic–pituitary–testicular axis [21,24,25,26]; an increase in pregnancy complications, a decrease in fertilization and live birth rates in natural conception and after IVF, and an impact on offspring development [8,19,27]. It is suggested that the main mechanisms by which obesity reduces semen quality may include systemic inflammation, oxidative stress, and sperm DNA fragmentation.

Beyond reproductive function, overweight and obesity affect other physiological systems. The associations between obesity and a wide range of diseases are well known; moreover, obesity has become a target in the prevention of multi-morbidity. It is known that obesity substantially increases the risk of diabetes mellitus, cardiovascular diseases, endocrine and metabolic disorders, sleep apnea, osteoarthritis, certain types of cancer, and a number of psychological problems, and it increases the risk of mortality associated with these diseases [13,15]. The most characteristic lipid metabolism disturbances observed in overweight and obesity include increases in triglycerides, total cholesterol, and low-density lipoprotein cholesterol, and decreases in high-density lipoprotein cholesterol [28,29,30]. Obesity is closely associated with increased production of leptin, a hormone that is secreted by adipocytes and affects the hypothalamic–pituitary–testicular system, determining the impact of obesity on male reproductive potential [25,31].

The most striking manifestation of hormonal changes in overweight or obese men is a decrease in serum total testosterone [22,32,33]. Testosterone, a primary male sex hormone produced by Leydig cells, plays a crucial role in reproductive processes in the male. However, obesity-related testosterone deficiency, also known as male hypogonadism, can lead to impaired spermatogenesis, reduced sperm production and subfertility [23,34]. Obesity-related testosterone deficiency associated with biochemical evidence (a cut-off level of total testosterone ≤ 12 nmol/L) and clinical symptoms (impaired sexual, physical or mental performance, low bone mineral density and others) is known by the term male obesity-related secondary hypogonadism (MOSH). There is substantial evidence indicating a strong association between MOSH and the development of a wide range of diseases. MOSH is considered an indicator of overall poor health and reduced quality of life [23].

Many factors of modern men’s lifestyles (obesity, physical inactivity, smoking, alcohol, and others) negatively affect fertility, making research on this problem relevant in both fundamental and applied aspects [10,35,36]. Physiological changes in the reproductive system of obese sedentary men remain underexplored; in particular, it is unclear which types of physical activity can improve semen quality and hormonal parameters [37]. Existing studies indicate inconsistency and ambiguity in results regarding the impact of a physically active or sedentary lifestyle on spermatogenesis and hormonal profile in obese men; therefore, the question of whether physical exercises improve male fertility in obesity remains a matter of debate [24,38,39]. Regarding Russian and Belarusian populations, there is very limited data on the impact of physical inactivity or sports activities on semen quality and levels of reproductive hormones, which are essential components of male fertility.

The aim of this study is to examine the relationship between abdominal obesity and sperm quality indicators, including hormonal and metabolic status, as well as to assess the impact of obesity-related testosterone deficiency and a sedentary lifestyle on anthropometric indicators, semen quality, and hormonal and metabolic parameters in young men from the general population. It is expected that the results will improve our understanding of the influence of obesity, obesity-associated testosterone deficiency and physical inactivity on male fertility indicators and will provide a broader perspective for further study in this area.

## 2. Materials and Methods

### 2.1. Study Population

This study was conducted on a population-based sample of men (n = 1283, median age 23.0 years) living in four cities: Arkhangelsk, Novosibirsk, Kemerovo (The Russian Federation) and Minsk (The Republic of Belarus). Minsk is located in the eastern part of Europe, Arkhangelsk in the subpolar zone of the European North of Russia, and Novosibirsk and Kemerovo in Western Siberia. The population of all four cities is predominantly Slavic (approximately 90–95%).

In all cities, the study design and standardized recruitment protocol were the same; they were described previously in detail [40,41]. In brief, the study enrolled male volunteers from the general population regardless of their fertility status. All participants were born or lived for at least 3–5 years in the cities where the study was conducted. At the time of the examination, the vast majority of men were employees or students of higher education institutions and had not previously undergone andrological examination. The inclusion criteria for participation consisted of the absence of acute general illnesses or chronic diseases in the acute phase, as well as the absence of urogenital infections. Each participant completed a standardized questionnaire, in which they indicated their age, place of birth, nationality, marital status, and certain lifestyle characteristics. The participants’ data were kept confidential. All participants gave written informed consent to participate in the examination.

All participants were examined by an experienced andrologist, and the examination results, in particular, current urogenital disorders, past acute illnesses, and current chronic diseases, were recorded. Before the physical examination, each participant was informed about the need to abstain from sexual activity for 2–7 days. For all participants, measurements of anthropometric parameters were performed (height, body mass, waist and hip circumference) and body mass index was calculated. The testicular volume was assessed using a Prader orchidometer and is presented as bitesticular volume (BTV). For each participant, a fasting morning blood sample was drawn before semen collection. Serum samples were stored at a temperature of approximately −40 °C until biochemical analyses were performed. Semen samples were collected by masturbation.

### 2.2. Determination of Abdominal Obesity

Clinical assessment of obesity includes several anthropometric markers—body mass index (BMI), waist circumference (WC), waist-to-hip ratio, and waist-to-height ratio—although more advanced methods such as magnetic resonance imaging and dual X-ray absorptiometry provide more detailed information about body composition and fat distribution [42,43]. In clinical practice or population-based studies, obesity screening tools should be simple, accessible, and reliable in terms of anthropometric indicators, such as BMI or WC. However, BMI has limitations in differentiating fat mass from muscle mass and in identifying abdominal fat. Unlike BMI, WC, as a surrogate indicator of abdominal obesity, can gauge the degree of AO, for example, in younger or older men and in athletes, where WC may differentiate the phenotypes associated with abdominal fat accumulation [44]. Thus, in our study, we used WC as a simple, fast, and adequate indicator of adiposity status, which is especially valuable in population-based studies.

### 2.3. Semen Analysis

Semen analysis was performed according to the WHO laboratory manual for the examination and processing of human semen [45,46] and has been described earlier [40,41]. Semen analysis included the measurement of semen volume, sperm concentration, a proportion of progressively motile and morphologically normal sperm, determination of the sperm DNA fragmentation index (DFI), and the total seminal zinc content. Sperm concentration was determined using a Goryaev hemocytometer under a light microscope after staining an aliquot of semen with trypan blue. The proportion of motile sperm with progressive linear movement and speeds greater than 25 and 2–25 µm/s (categories A and B, respectively) was assessed using the SFA-500-2 semen analyzer (Biola, Moscow, Zelenograd, Russia). Morphological analysis of spermatozoa was performed in accordance with WHO recommendations [46]. Semen smears were stained with Diff-Quick kits (Abris+, Saint-Petersburg, Russia). The first 200 spermatozoa were analyzed under a light microscope Axio Scope.A1 (Carl Zeiss, Oberköchen, Germany) at a magnification of ×1000 with oil immersion. Sperm dimensions were measured using an ocular micrometer. The morphology of spermatozoa was studied twice in a random, blinded order by a trained staff member.

Sperm DNA fragmentation was assessed through the SCSA method (sperm chromatin structure assay) using flow cytometry as proposed by Evenson [47] with a small modification described earlier [35]. Briefly, immediately after obtaining semen, an aliquot of 300 μL was frozen and stored at −40 °C; thawing was not allowed before analysis. For the DNA fragmentation analysis, the sample was subjected to rapid thawing and diluted in TNE buffer to a sperm concentration of 1 × 10^6^/mL. To 100 μL of the diluted sperm, 200 μL of acidic buffer was added. After incubation for 30 s, 600 μL of staining solution was added, containing 6 mg/L acridine orange. No more than one hour later, the number of spermatozoa with red and green fluorescence was counted using a Guava EasyCyte Mini fluorescence cytometer (Luminex Corporation, Austin, TX, USA). Each sample was evaluated three times, at 5000 cells per run. The DFI was calculated as the fraction of cells with red fluorescence (DNA-fragmented sperm) relative to the total number of cells with red and green fluorescence. No DNA fragmentation analysis was performed in semen samples obtained from azoospermic and severe oligozoospermic men.

### 2.4. Analysis of Reproductive Hormones, Zinc, and Metabolites

Serum concentrations of luteinizing hormone (LH), follicle-stimulating hormone (FSH), total testosterone, estradiol, inhibin B, and leptin were determined through an immunoassay using commercially available kits according to the manufacturer’s instructions (Alkor Bio, Hema, Moscow, Russia; Beckman Coulter, Brea, CA, USA; Diagnostics Biochem Canada Inc., London, ON, Canada). The measurement range for testosterone was 0.2–50 nmol/L with a sensitivity of 0.2 nmol/L; oestradiol 0.1–20 nmol/L with a sensitivity 0.025 nmol/L; FSH 2.0–100 mIU/mL with a sensitivity 0.25 mIU/mL; LH 2.0–90 mIU/mL with a sensitivity 0.25 mIU/mL; inhibin B 12–105 pg/mL with a sensitivity 2.6 pg/mL; and leptin 1–100 ng/mL with a sensitivity 0.5 ng/mL.

The serum and seminal zinc concentration was determined through spectrophotometric and direct colorimetric methods without deproteinization using commercial kits according to the manufacturer’s instructions (Vital Development Corporation, Saint Petersburg, Russia). A detailed description of the method has been given previously [35,40]. Serum concentrations of triglycerides (TG), total cholesterol (TC), high-density lipoprotein cholesterol (HDL-C), glucose, and uric acid were determined through the enzymatic colorimetric method in a plate-based modification using commercial kits according to the accompanying instructions (Vector Best, Novosibirsk, Russia). Low-density lipoprotein cholesterol (LDL-C) was determined through calculation using Friedewald’s formula [48]. The measurement range for TG was up to 11.4 mmol/L; TC up to 27 mmol/L; HDL-C up to 3.0 mmol/L; glucose up to 28.0 mmol/L; and uric acid up to 1500 μmol/L.

### 2.5. Statistical Analysis

**Figure 1:**
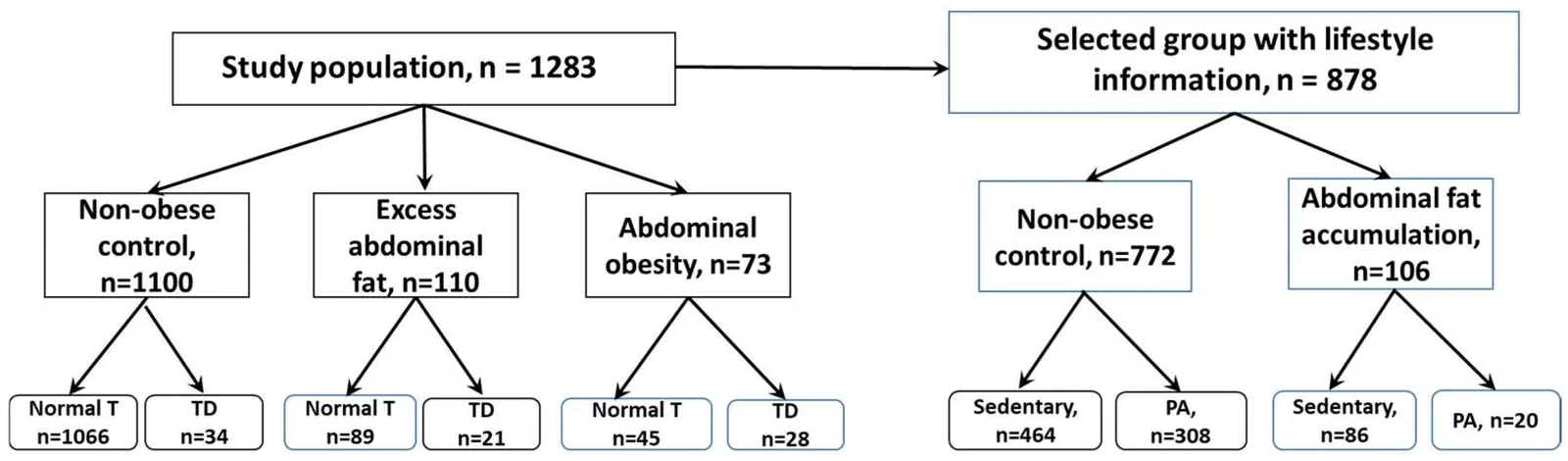
A schematic representation of the general research plan. Note: T—testosterone; TD—testosterone deficiency; PA—physical activity; additional description in the text.

To identify a relationship between AO and anthropometric, semen, hormonal, zinc and metabolic parameters, the study population was stratified into three groups by WC according to the WHO guidelines [12]: non-obese control (WC < 94 cm); excess abdominal fat (94 cm ≤ WC < 102 cm); and AO (WC ≥ 102 cm). Comparisons of anthropometric, semen, hormonal, zinc, and metabolic parameters were performed between these three groups. A schematic representation of the overall research design is shown in Figure 1.

To assess the relationship between testosterone deficiency (TD) and anthropometric, semen, hormonal, zinc, and metabolic indicators in participants with different obesity statuses, each of the three WC groups was stratified into two subgroups based on the testosterone level: the TD subgroup (T ≤ 12.1 nmol/L) and the normal testosterone subgroup (T > 12.1 nmol/L). Since clinical symptoms of androgen deficiency were not systematically evaluated in this study, here and below, we will use the term “biochemical testosterone deficiency” or “biochemical TD”. The subgroup designations and the number of participants in each of them are shown in Figure 1.

In order to analyze anthropometric, semen, hormonal, zinc, and metabolic changes in men with different lifestyles (physically active or sedentary), part of our study population (n = 878) was additionally surveyed about the special characteristics of the participant’s profession (mental, mental/physical and physical labor) and their involvement in recreational sports, including the type and frequency of physical exercises. Participants were also asked to provide a general self-assessment of their lifestyle over the past year as sedentary or physically active. As a definition of a sedentary lifestyle, participants were asked to sum up their proportion of physical labor related to professional activity, recreational sports activities, and daily sitting (≥4–6 h) without additional physical exercises. The participants were initially stratified into two groups: the non-obese control group (WC < 94 cm) and the abdominal fat accumulation group (WC ≥ 94 cm). The latter group combined participants with excess abdominal fat and AO. Subsequently, based on these personal data, each group was divided into two subgroups: the first included sedentary men, and the second included physically active men (Figure 1). Comparisons of anthropometric, semen, hormonal, zinc and metabolic parameters were performed between the sedentary subgroup and the physically active subgroup, both among non-obese control men and among those with abdominal fat accumulation.

Statistical data processing was performed using the STATISTICA software package (version 8.0). Normality of distribution for semen, hormonal, and metabolic parameters was assessed using the Kolmogorov–Smirnov test. Parameters which were not normally distributed were transformed using an arcsine transformation (progressive motility, DFI), square root transformation (semen volume, total sperm count, sperm concentration, testosterone, oestradiol and inhibin B levels), or logarithmic transformation (body weight, BMI, WC, hip circumference, BTV, the levels of LH, FSH, leptin, TG, TC, HDL-C, LDL-C, glucose, uric acid, serum zinc levels and seminal zinc contents) before analysis. Height and the proportion of morphologically normal sperm did not require adjustment. A one-way or two-way analysis of covariance (ANCOVA) was applied to identify differences in anthropometric, semen, hormonal, and metabolic indicators between groups. The categorical predictors (factors) in ANCOVA were the degree of abdominal fat accumulation (non-obese, excess abdominal fat, abdominal obesity), the serum testosterone level (normal testosterone or TD), and lifestyle (sedentary or physically active), with age, smoking, alcohol consumption, and sexual abstinence duration being considered as covariates.

Using the Chi-squared (χ^2^) test, a comparison was made of the TD frequency in three WC groups, as well as the frequency of a sedentary lifestyle in the non-obese control group and in the abdominal fat accumulation group. For pairwise group comparisons, Duncan’s test was applied. A *p* value < 0.05 was considered statistically significant. Descriptive statistics are presented using untransformed data. In the tables, all measured parameters are presented as means (SD) and medians (5–95 percentiles). Spearman correlation coefficients were used to determine correlations among selected parameters.

## 3. Results

### 3.1. General Information About the Entire Study Population

The mean age of the participants was 25.1 years (median age 23.0 years). The majority of the study population consisted of young men—85.8% (age 18–30 years). The ethnic composition was as follows: Slavs, 86.0%; descendants of mixed marriages, 12.2%; other ethnicities, 1.8%. Of the total number of participants, 30.7% were married, 9.3% had children, 28.0% smoked, and 74.3% consumed alcohol. At the time of examination, 15.2% of the men had reproductive system diseases, including chronic prostatitis, varicocele, cysts of the testis or epididymis, and hydrocele.

Of the total number of participants, 98.0% provided a semen sample. Azoospermia was detected in 1.5% and pathozoospermia in 33.3%; the remaining 65.2% were characterized as normozoospermia, i.e., having a sperm concentration ≥ 16.0 million/mL, a proportion of sperm with progressive motility ≥ 30%, and a proportion of sperm with normal morphology ≥ 4.0%, in accordance with the reference values of the norm [45,46].

Participants with congenital malformations of the reproductive organs (hypospadias, cryptorchidism or consequences of cryptorchidism surgery), the carriers of AZF deletions (b2/b4), those who took anabolic steroids (self-reported or indicated in the profile of reproductive hormones), and men with missing WC were excluded. Thus, a total of 1283 named men from the general population were included in our study population.

### 3.2. Comparison of Anthropometric, Seminal, Hormonal, Zinc and Metabolic Parameters Between Participants with Different Abdominal Fat Accumulation

In our study population, the prevalence of AO was 5.7%, and the prevalence of excess abdominal fat was 8.6%; the remaining 85.8% served as the non-obese control group. Men with excess abdominal fat or AO were significantly older compared with non-obese men (*p* < 0.05, Table 1). There were significant differences in anthropometric parameters between the three WC groups. Body weight, BMI, WC and hip circumference were significantly higher in the AO group compared with the non-obese control (*p* < 0.05, Table 1). The group with excess abdominal fat occupied an intermediate position in anthropometric measures but differed significantly from both the non-obese control and AO groups (*p* < 0.05).

A significant reduction in semen parameters was observed in the AO group compared with the non-obese control group (*p* < 0.05), with the exception of sexual abstinence, BTV and the proportion of morphologically normal sperm, which did not differ among them (Table 1). Semen volume, total sperm count, sperm concentration, and progressive motility were significantly reduced in the AO group compared to the non-obese control group (*p* < 0.05), but these parameters did not differ between the excess abdominal fat group and the non-obese control group. Notably, the sperm DFI increased significantly in both the excess abdominal fat group and the AO group compared with the non-obese control group (*p* < 0.05). The data indicate a substantial decline in semen indicators among young men with AO.

The most pronounced significant differences between WC groups were observed for testosterone and leptin levels (Table 1). The excess abdominal fat and AO groups were characterized by a significant, graded decrease in testosterone and an increase in leptin levels compared to the non-obese control group (*p* < 0.05). The FSH level was significantly higher, while inhibin and estradiol levels were significantly lower in the AO group compared to the non-obese control group (*p* < 0.05). Changes in the reproductive hormonal profile reflect impaired function of Leydig and Sertoli cells in men with AO, which intensifies as abdominal fat mass increases and, consequently, leptin production by adipocytes increases.

In the excess abdominal fat group and the AO group, biochemical markers of metabolism showed a significant graded increase compared to the non-obese control group (serum levels of TG, glucose, and uric acid; *p* < 0.05, Table 1). Note that the mean levels of TG and uric acid in the AO group exceeded the reference values of the norm (≤1.7 mmol/L and ≤420 μmol/L, respectively). The AO group differed from the non-obese control group in having higher levels of TC, HDL-C, and LDL-C (*p* < 0.05, Table 1). Although the metabolic indicators in participants with excess abdominal fat were less pronounced than in the AO group, they were significantly higher than in the non-obese control group (*p* < 0.05, Table 1). In addition, the AO group showed increased serum zinc levels and a reduced semen zinc content compared to the non-obese control group (*p* < 0.05, Table 1). Disturbances in the metabolic profile of men with various abdominal fat accumulations indicate worsening metabolic health.

Correlation analysis revealed that WC was significantly correlated with BMI (r = 0.84; *p* < 0.05), hip circumference (r = 0.81; *p* < 0.05), leptin level (r = 0.61; *p* < 0.05) and almost all metabolic parameters (for TG r = 0.45; for OC r = 0.33; for LDL-C r = 0.35; for glucose r = 0.28; for uric acid r = 0.39; *p* < 0.05 for all cases). A strong inverse relationship was observed between WC and serum testosterone levels (r = −0.39, *p* < 0.05), but a strong positive correlation was found between WC and serum zinc levels (r = 0.36; *p* < 0.05). No significant correlation coefficients were found between sperm quality parameters and the lipid profile. There were inverse but very weak relationships between WC and all semen indicators, including DFI (r value ranged from −0.08 to −0.21; *p* < 0.05).

### 3.3. Comparison of Anthropometric, Seminal, Hormonal, Zinc, and Metabolic Parameters in Participants with Different Degrees of Abdominal Fat Accumulation Associated with Testosterone Deficiency

The frequencies of participants with different testosterone levels (biochemical TD or normal levels) were highly significantly (χ^2^_2_ = 171.5, *p* < 0.0001) dependent on their degree of abdominal fat accumulation (non-obese control, excess abdominal fat, abdominal obesity). The biochemical TD was detected in 38.7% of AO participants, 19.5% of excess abdominal fat participants, and 3.2% of non-obese participants (χ^2^_2_ = 160.4, *p* < 0.0001). Thus, the TD prevalence varied significantly across different WC groups, indicating an association between TD extent and abdominal fat accumulation.

The relationship between biochemical TD and anthropometric indicators was weak across all WC groups (Table 2). In the non-obese control group, the biochemical TD was associated with an increase in WC (*p* < 0.05, Table 2), but this was not observed in the groups with excess abdominal fat or AO. It is worth noting that the non-obese control group with normal testosterone levels was significantly younger than the other WC groups (*p* < 0.05, Table 2). This suggests that age may be one of the reasons for abdominal fat accumulation.

In all WC groups, biochemical TD was not related to BTV and semen quality, including semen volume, total sperm count, sperm concentration, motility and normal morphology (Table 2). In the AO group, biochemical TD was associated with a significant increase in DFI as well as FSH and leptin levels (*p* < 0.05, Table 2), but biochemical TD was not related to the levels of LH, estradiol, and inhibin B in all WC groups (Table 2).

In the AO group, biochemical TD was significantly associated with elevated levels of TG, TC, LDL-C, glucose, and uric acid (*p* < 0.05), while the levels of TG, LDL-C, glucose, and uric acid exceeded the reference values of the norm (≥1.7 mmol/L; ≥3.0 mmol/L; ≥6.1 mmol/L; ≥420 μmol/L, respectively) (Table 2). Thus, obesity-associated TD was related to a further weakening of metabolic health, which had already been disrupted by AO.

As Table 2 shows, biochemical TD was observed together with elevated serum zinc levels in all WC groups (*p* < 0.05); the most significant increase was observed in the AO group. At the same time, biochemical TD was not associated with seminal zinc content in all WC groups.

### 3.4. Comparison of Anthropometric, Seminal, Hormonal, Zinc, and Metabolic Parameters Between Participants with Abdominal Fat Accumulation Associated with a Sedentary or Physically Active Lifestyle

Since some of the participants in our study population provided additional information on the nature of their professional labor, the frequency and intensity of their recreational sports, and a self-assessment of their lifestyle (sedentary or physically active lifestyle), it became possible to evaluate the relationship between certain lifestyles and anthropometric, semen, hormonal and metabolic indicators (Figure 1). After dividing this cohort of participants into four independent subgroups based on WC and extent of PA, a comparative analysis revealed the following results (Table 3). In the cohort with abdominal fat accumulation, body weight, BMI and WC were significantly higher in the SB subgroup compared with the PA subgroup (*p* < 0.05, Table 3). In the same cohort, there were no differences between the SB and PA subgroups in height, hip circumference, BTV, sexual abstinence, DFI and other semen parameters, except for semen volume, which was significantly higher in the PA compared with the SB subgroup (Table 3). In obese men, a physically active lifestyle showed a more favorable hormonal profile: LH and testosterone levels were significantly higher, and leptin levels were significantly lower in PA men compared with SB ones (*p* < 0.05). In obese men, a physically active lifestyle also showed significantly improved metabolic and zinc profiles: TG, TC, LDL-C, uric acid and zinc levels decreased in the PA subgroup compared with the SB subgroup (*p* < 0.05, Table 3). Among non-obese participants, no differences were found between the subgroups with sedentary and physically active lifestyles across all the studied indicators (Table 3).

The frequencies of participants with different degrees of PA (sedentary or PA lifestyle) were highly significantly (χ^2^_1_ = 17.8, *p* < 0.0001) dependent on their degree of abdominal fat accumulation (non-obese control and abdominal fat accumulation). A sedentary lifestyle was detected in 81.0% of abdominal fat accumulation participants and in 59.6% of non-obese participants (χ^2^_1_ = 6.7, *p* < 0.01).

## 4. Discussion

In this study, a multi-faceted analysis of the consequences of AO was conducted in young men, integrating clusters of anthropometric, semen, hormonal, and metabolic indicators. The main conclusion is that abdominal obesity is accompanied by a decrease in semen quality, including increased sperm DFI, a dysregulation of the hypothalamic–pituitary–testicular axis, and adverse changes in the metabolic profile. Abdominal obesity associated with TD was accompanied by further adverse changes in sperm DNA integrity, as well as in the levels of reproductive hormones and metabolites. Among men with abdominal obesity who lead a physically active lifestyle, a more favorable hormonal and metabolic profile was observed compared with those who lead a sedentary lifestyle.

Excess body weight and obesity are the most important lifestyle factors and key risk factors for reproductive health [7,8,9,25,26,38]. In recent decades, a behavioral pattern has formed in the global community that has contributed to the rising number of people suffering from obesity, which can rightly be regarded as a disease of civilization. Modern social behavior includes a general decrease in physical activity and the spread of a sedentary lifestyle, which is due to the automation of labor, widespread use of transportation, primarily private cars, and consumption of enhanced energy-dense foods, including fast food containing large amounts of refined fats and easily digestible carbohydrates, as well as a high level of psychological stress among people living in megacities [10,39,49,50]. The prevalence of obesity among men is rapidly increasing worldwide; of particular concern is the growing AO among young people, who make up the most active reproductive part of our society [11]. The results of our study demonstrate that, in young men from the general population, greater abdominal adiposity was associated with less favorable semen quality indicators and hormonal and metabolic profiles, increasing the risk of infertility and reducing the chances of conception. These results are consistent with the experimental data of other researchers and the conclusions of recent meta-analyses [10,17,18,19,22,24,26,51,52,53,54,55].

Semen analysis is a crucial step in evaluating men, providing information about semen quality; however, it generally does not adequately assess fertility. Even normal semen quality indicators (concentration, motility, normal morphology) do not exclude clinical male factor infertility, since they do not reflect the sperm’s fertilizing ability, nor the integrity of their DNA. The assessment of sperm DNA fragmentation can provide additional and independent information about semen quality, paternal genome integrity, and fertility status [9,47,56,57]. Our study revealed an increased level of sperm DNA fragmentation in participants with excess abdominal fat or AO, which, as is already known, can have a negative impact on embryo development, reproductive functions, and overall health of the offspring [56]. The molecular mechanism of disruption of sperm DNA integrity includes several processes, among which incomplete protamination during spermiogenesis can be highlighted, as well as the impact of reactive oxygen species (ROS, oxidative stress) during functional sperm maturation in the epididymis, leading to single-strand and double-strand DNA breaks [17,56,57]. In obesity, abdominal adipose tissue produces an increased amount of proinflammatory cytokines, which intensifies systemic oxidative stress, leading to increased generation of ROS, which induces sperm DNA damage. This process is also facilitated by prolonged exposure to scrotal hyperthermia, increasing testicular temperature and reducing semen quality and sperm DNA integrity in AO men [6,10,56].

Zinc is one of the most biologically important trace elements for the male reproductive system [58,59,60,61]. Seminal zinc determination is considered a useful tool in addition to other parameters in assessing male reproductive parameters, since it is positively associated with semen quality [35,58,60]. In our study, we found that AO men compared with non-obese men had a lower seminal zinc content, which coincides with their reduced semen quality, although neither the sperm concentration nor the zinc seminal content falls below the accepted reference values of the norm (for sperm concentration, it is ≥16.00 million/mL; for zinc content, ≥2.4 µmol/ejaculate). As a structural and catalytic cofactor of many enzymes, zinc exhibits antioxidant, antibacterial, and anti-apoptotic properties in the testes [59]; thus, a reduced seminal zinc content in AO men may reflect a decrease in the antioxidant protection of the testes. In contrast to the semen zinc content, the association between serum zinc levels and semen quality remains controversial, since some researchers have failed to find a positive relationship between serum zinc concentrations and semen parameters [35,62,63].

Our study clearly demonstrates a close association between abdominal adiposity and the reproductive hormonal profile, which is primarily due to a decrease in testosterone and inhibin B levels and an increase in FSH levels, indicating a dysregulation of the hypothalamic–pituitary–testicular axis and the impaired function of Leydig and Sertoli cells. Our data are in line with the results of other studies and recent reviews [10,25,26,55]. Under physiological conditions, the hypothalamic–pituitary–testicular axis is activated by kisspeptins through the regulation of GnRH secretion, which stimulates the release of pituitary LH and FSH. LH stimulates testosterone secretion by Leydig cells and together with FSH supports proper spermatogenesis [9,24,25,33,64]. Thus, the interactions described above within the hypothalamic–pituitary–testicular axis may partially clarify the relationship between excessive fat accumulation and the deterioration in semen quality.

The results of our study demonstrate an increase in TG, TC, and LDL-C levels, as well as a decrease in semen quality in AO men. These findings suggest a close relationship between lipid profile and spermatogenesis. Although it has been firmly established that obesity is associated with changes in the lipid profile, the nature and mechanisms of these changes are not fully understood [28,30,65]. The potential importance of lipids for male reproductive function is due to the fact that cholesterol is a primary source for steroid biosynthesis in the testes [66]. Although Leydig cells are capable of synthesizing cholesterol *de novo* from acetate, plasma lipoproteins remain the main source of steroid biosynthesis. Lipid metabolism, including fatty acid oxidation, is essential for energy homeostasis in Sertoli cells [36,67]. Sertoli cells are also capable of synthesizing cholesterol *de novo* from acetate, which is necessary for spermatogenesis, but this synthesis is insufficient, so cholesterol must also be supplied from the bloodstream. Lipids are also needed for remodeling the membranes of developing germ cells, since the plasma membrane of spermatozoa has a double lipid layer; therefore, cholesterol homeostasis is crucial for post-testicular sperm maturation in the epididymis, where the sperm membrane becomes more fluid. Imbalances in cholesterol levels can particularly disrupt these processes [65].

Studies on the relationship between serum lipid levels and sperm parameters have yielded conflicting results, as they have failed to identify a relationship between serum lipid levels and semen quality, including the present study [28,41,68]. However, seminal lipid levels (TG, TC, HDL-C, and LDL-C) were negatively correlated with semen parameters (semen volume, total sperm count, sperm concentration and progressive motility). In addition, the seminal levels of TG, TC, HDL-C, and LDL-C were higher in patients with oligozoospermia, asthenozoospermia, and teratozoospermia than in patients with normal sperm concentration, motility, or morphology [69]. So, it stands to reason that if the seminal lipid level reflects the semen quality indicators, then lipid metabolism disorders in the male reproductive system may contribute to the development of male subfertility. Furthermore, it is known that lipid metabolism disorders associated with obesity can play a certain role in the development of chronic inflammation, cardiovascular diseases, insulin resistance, and metabolic syndrome [13,29,70].

An important part of this study was examining the relationship between biochemical TD and indicators of reproductive health, both as an independent factor and in association with AO. The primary laboratory method for diagnosing TD is serum testosterone concentration ≤ 12.1 nmol/L [23,71]. In our study, biochemical TD was detected in 38.7% of obese men and in 3.2% of non-obese men. According to the results of other epidemiological studies, TD was observed in 24.1% of men in the USA general population aged 20–40 years [30] and in 2.1–12.8% of men in the European general population [72]. However, other authors report higher prevalence rates of TD: 32% of adult men without obesity and 75% of men with severe obesity (BMI > 40 kg/m^2^) [33]. The prevalence of TD was higher among clinical groups with metabolic diseases (type 2 diabetes, obesity, metabolic syndrome) and reached 78.8% [72].

Evidence from our study indicates that obesity-associated biochemical TD is accompanied by increased sperm DNA fragmentation and elevated levels of FSH, leptin and zinc, as well as TG, TC, LDL-C, glucose, and uric acid levels, compared to obese men with normal testosterone levels. The molecular mechanism underlying the development of obesity-associated functional TD is linked to an imbalance in the hypothalamic–pituitary–testicular axis, initiated by increased production of adipokines by abdominal and visceral adipose tissue [73]. At physiological concentrations, adipokine leptin stimulates hypothalamic neurons and GnRH secretion via the kisspeptin pathway, which increases pituitary LH secretion and promotes testosterone production. Testosterone enhances the activity of lipoprotein lipase enzyme, leading to triglyceride uptake into adipocytes. However, AO is very often accompanied by hypothalamic leptin resistance, which ultimately leads to reduced testosterone production [9,23]. Moreover, leptin can act directly or indirectly through receptors in testicular tissue, suppressing the action of gonadotropins on Leydig cells, exacerbating TD. In addition, adipocytes exhibit increased aromatase expression, increasing the conversion of testosterone to estradiol and thereby reducing the level of circulating androgens, while higher estrogen levels via negative feedback inhibit the secretion of GnRH and LH, further reducing testosterone levels [23,74]. In our study, no rise in the estradiol level was detected; rather, a small decrease in its peripheral circulation was observed in AO men in comparison with non-obese ones, suggesting the limited role for estradiol in the imbalance of the hypothalamic–pituitary–testicular axis in AO individuals. Other researchers have found elevated estradiol levels only in patients with morbid obesity [73].

The determination of total body fat percentage, abdominal fat percentage, and pelvic fat percentage by densitometry revealed a negative correlation between fat content in different body regions and testosterone levels [55]. These data are consistent with our findings obtained when assessing the amount of abdominal fat using WC as a surrogate anthropometric indicator. In our study, WC was positively correlated with leptin levels and negatively with testosterone levels, supporting the view that increased leptin production by adipocytes may be a factor determining TD in AO men.

It is interesting to note that obesity-associated biochemical TD was not related to semen quality indicators, with the exception of sperm DNA fragmentation, despite a substantial decrease in serum testosterone levels. This discrepancy may be due to an as yet unexplained fact: spermatogenesis is supported by exclusively intra-testicular testosterone, and its intra-testicular concentration is more than 100 times higher than in the peripheral circulation of adult men [34]. Intra-testicular testosterone is produced by Leydig cells and regulates the process of spermatogenesis, influencing the meiosis of spermatocytes, spermiogenesis, maintenance of the blood–testis barrier, and the growth of Sertoli cells [64]. Thus, the level of circulating testosterone does not reflect its testicular content, it cannot be considered a marker of spermatogenesis, and it cannot be associated with pathological changes in semen quality in adult men. Currently, there are no data on the reference values for intra-testicular testosterone concentrations required for normal human spermatogenesis.

We have demonstrated that serum lipid levels (TG, TC, LDL-C) were significantly higher among obese men with biochemical TD than among obese men without TD, and they often exceeded the reference values of the norm. Other indicators of metabolic health (glucose, uric acid) showed similar increases in obese men with biochemical TD. Thus, our data suggest a relationship between lipid metabolism and androgen status in men with abdominal adiposity. Some authors believe that, in obese men with TD, the relationship between testosterone and metabolic status is bidirectional and forms a vicious circle [33]. Testosterone deficiency is linked to fat accumulation, and the abnormal expansion of adipose tissue disrupts testosterone production, leading to further fat accumulation. However, under physiological conditions, testosterone makes a significant contribution to the regulation of lipid metabolism when it suppresses fat accumulation by binding to androgen receptors in abdominal adipose tissue and inhibiting lipoprotein lipase activity, thereby promoting lipolysis and preventing lipid accumulation [25,73].

It should be noted that TD can also be accompanied by a clinical syndrome, the main manifestations of which are erectile dysfunction and, consequently, infertility [9,20], general asthenia, gynecomastia, anemia, decreased muscle mass and strength, the deterioration of bone health, and reduced quality of life [28]. However, the functional nature of obesity-related male hypogonadism suggests that this condition is potentially reversible and can be treated by managing AO through dietary restrictions, pharmacotherapy, bariatric surgery, and testosterone replacement therapy [17,22,33,53,71]. The effectiveness of such therapeutic approaches is considered proven.

It is considered an indisputable fact that lifestyle significantly affects men’s health and male reproductive parameters. We discovered that 81.0% of obese men lead a sedentary lifestyle and do not engage in physical exercise in their free time. The results of our study also showed that, in obese people, a sedentary lifestyle was associated with additional accumulation of abdominal fat, an increase in leptin, metabolite and zinc levels, and lower levels of LH and testosterone compared with a physically active lifestyle. Thus, physically active men with abdominal obesity had a relatively more favorable hormonal and metabolic profile than sedentary obese men. The data obtained are consistent with the conclusions of other authors presented in the following reviews [36,38,49] showing an increase in testosterone and LH levels in obese men after physical training, which, in their opinion, may influence spermatogenesis and semen quality. Our data are also in agreement with the results of other studies, suggesting a weakening of metabolic health in the context of obesity combined with a sedentary lifestyle [36,37,49,75]. Furthermore, AO combined with a sedentary lifestyle is considered one of the main key risk factors for dyslipidemia, atherosclerosis, cardiovascular diseases, type 2 diabetes, and osteoarthritis [7,8,38,39]. In a recent meta-analysis, it was convincingly shown that weight loss resulting from interventions (diet and physical activity) led to an improved lipid profile (decreased TG, LDL-C and increased HDL-C levels) after 3–6 months of training [70]. Thus, changing a sedentary lifestyle can be an important step towards improving hormonal and metabolic status in obese men.

Although most studies indicate no substantial influence of recreational PA on semen quality, moderate PA can increase sperm concentration and motility, whereas intensive PA or certain sports (for example, cycling) can negatively affect semen quality [36,38,49]. The molecular processes underlying the changes induced by PA are caused by alterations in ROS production both systemically and in testicular tissue. Model animal studies have shown that high-intensity running or swimming leads to increased ROS production and lipid peroxidation, together with a decrease in enzymatic antioxidant activity [49]. A study by Iranian authors convincingly demonstrated that, in infertile men, resistance training for 24 weeks improved semen quality parameters and reduced sperm DNA fragmentation [76]. Moreover, resistance training also reduced markers of oxidative stress and inflammation in the semen of infertile men, while enhancing antioxidant defense.

It should be acknowledged that PA can serve as an additional treatment method that could improve hormonal and metabolic profiles and overall health in obese men, but there is no unified clinical consensus regarding the type, intensity, and effectiveness of physical activity due to conflicting research results. A review of 92 studies [37] found that, in obese men, fat deposits decreased during aerobic and resistance training lasting up to six months if energy intake was restricted. Chinese researchers performed a similar meta-analysis of 77 studies [77]. The analysis showed that, in obese adults, aerobic exercise yields the best results for reducing body weight and BMI, while high-intensity interval training is likely the best means to improve body composition (reducing WC and fat mass) and overall metabolic status (reducing triglyceride and glucose levels).

There are a few strengths and limitations to our study. The study’s major strength is its large sample size of young volunteers from the general population who were not selected based on body composition or semen quality, so our study population was fully representative of the young part of the general population. In addition, all our participants belonged to the same socio-cultural environment and ethnic community. A standardized recruitment protocol, questionnaire, and laboratory methods with the same equipment and supplies were used. Our study has a number of limitations, which are largely related to the specific features of population-based research and some of our methodological approaches, including the cross-sectional design and the difficulties in determining causal relationships. Firstly, our study population included very few obese participants, which could be explained by the fact that our study population mainly consisted of healthy young people, reflecting the situation in the general population. This population-specific feature also explains the substantial age-related differences between obesity groups, as fat accumulation is mainly a consequence of age-related lifestyle factors (unhealthy diet and physical inactivity). Secondly, lifestyle information was obtained by self-reporting, leaving a chance of misclassification. Self-reporting information is used in most epidemiological studies, including our study. Although the results of our comparative study allow for a fairly clear assessment of male reproductive parameters, as well as the hormonal and metabolic status in sedentary obese men, to further confirm the positive relationship between PA and men’s reproductive health, a larger sample size and more controlled methods of measuring PA will be required. It would be desirable to conduct an interventional study in which the same group of sedentary obese men is subjected to regular controlled PA of varying intensity and duration. The results would provide additional information on how a sedentary lifestyle weakens the markers of reproductive and metabolic health and how PA yields positive outcomes. Thirdly, we used WC as a surrogate rather than a direct measurement of abdominal adiposity. In population-based studies as well as in clinical practice, assessment of obesity includes BMI or WC as anthropometric markers, although more advanced and more expensive and labor-intensive methods are proposed to obtain more detailed information about body composition and fat distribution. Population-based studies must be conducted on a large study population, which dictates the choice of appropriate survey methods due to the large number of samples requiring quick and reliable results. As a result, we chose WC as a simple, fast, and adequate indicator of abdominal adiposity, which is especially valuable in population-based studies.

## 5. Conclusions

In men from the general population, a relationship was found between abdominal fat accumulation and sperm concentration and motility, sperm DNA fragmentation, and changes in hormonal and metabolic status. Therefore, greater abdominal adiposity was associated with a less favorable semen quality and hormonal and metabolic profile. The obesity-associated biochemical TD was accompanied by a further increase in sperm DNA fragmentation, as well as elevated levels of FSH, leptin, lipids, glucose, uric acid, and zinc. This suggest that biochemical TD identified an obesity subgroup with particularly unfavorable hormonal and metabolic characteristics. In physically active obese men, the hormonal and metabolic profile was relatively more favorable than in obese men who led a sedentary lifestyle. Whether modification of PA or abdominal adiposity directly improves reproductive, hormonal and metabolic parameters should be investigated prospectively.

Thus, we can conclude that abdominal obesity, as well as abdominal obesity associated with biochemical TD or a sedentary lifestyle, is a potential risk factor for impaired reproductive and metabolic health. Given that abdominal fat accumulation is associated with unfavorable semen quality and hormonal and metabolic status, the clinical assessment of men’s reproductive health should, as a first step, include measuring WC as a simple and informative surrogate indicator of AO, as well as the level of total testosterone as an important indicator of hormonal status. The data obtained highlight the importance of maintaining a healthy body composition for preserving active male reproductive function.

## Figures and Tables

**Table 1:** Anthropometric, semen, hormonal, zinc and metabolic indicators of participants with different abdominal fat accumulation.

Variable	Non-Obese Control (n = 1100) (WC < 94 cm)	Excess Abdominal Fat (n = 110) (102 > WC ≥ 94 cm)	Abdominal Obesity (n = 73) (WC ≥ 102 cm)
Age, years	24.1 ± 6.0 ^a^ 22.0 (18.0–35.0)	30.2 ± 8.4 ^b^ 28.5 (20.0–45.0)	32.0 ± 9.4 ^c^ 31.0 (20.0–51.0)
Body weight, kg	74.7 ± 9.4 ^a^ 74.0 (60.0–90.3)	93.4 ± 8.1 ^b^ 93.0 (80.0–105.9)	106.1 ± 11.1 ^c^ 105.0 (90.0–129.0)
Height, cm	179.0 ± 6.7 ^a^ 179.0 (169.0–190.0)	181.6 ± 7.0 ^b^ 182.0 (169.0–193.0)	181.0 ± 7.0 ^b^ 181.0 (169.5–193.0)
BMI, kg/m^2^	23.3 ± 2.6 ^a^ 23.2 (19.2–27.7)	28.3 ± 2.3 ^b^ 28.2 (25.2–32.0)	32.4 ± 3.1 ^c^ 31.5 (27.9–38.3)
Waist circumference, cm	80.4 ± 6.3 ^a^ 80.0 (70.0–91.0)	97.0 ± 2.3 ^b^ 97.0 (94.0–101.0)	107.6 ± 5.4 ^c^ 106.0 (102.0–120.0)
Hip circumference, cm	96.2 ± 6.0 ^a^ 96.0 (87.0–106.0)	106.8 ± 4.9 ^b^ 106.0 (99.0–114.0)	113.3 ± 5.7 ^c^ 112.0 (106.5–126.0)
Sexual abstinence, days	4.6 ± 3.7 ^a^ 4.0 (2.0–8.0)	4.4 ± 2.7 ^a^ 4.0 (2.0–7.0)	4.1 ± 2.3 ^a^ 4.0 (2.0–7.0)
BTV, mL	41.8 ± 8.4 ^a^ 40.0 (27.0–50.0)	45.6 ± 8.0 ^b^ 48.0 (30.3–60.0)	42.6 ± 10.1 ^a^ 40.0 (25.0–60.0)
Semen volume, mL	3.8 ± 1.7 ^a^ 3.6 (1.3–6.8)	3.7 ± 1.8 ^a^ 3.5 (1.3–6.6)	3.0 ± 1.5 ^b^ 2.8 (0.9–5.5)
Total sperm count, ×10^6^/ejaculate	251.2 ± 223.0 ^a^ 204.1 (14.7–673.1)	215.4 ± 205.6 ^a^ 166.6 (7.6–539.8)	140.5 ± 129.7 ^b^ 106.7 (0.0–411.7)
Sperm concentration, ×10^6^/mL	67.47 ± 52.28 ^a^ 55.23 (5.50–167.00)	61.49 ± 52.56 ^a^ 45.09 (4.37–173.14)	46.17 ± 39.60 ^b^ 34.48 (0.0–144.01)
Progressive motility, %	48.8 ± 26.8 ^a^ 49.2 (4.3–90.9)	45.0 ± 29.3 ^a^ 41.0 (3.1–91.6)	37.2 ± 27.5 ^b^ 33.9 (1.6–86.7)
Normal morphology, %	7.04 ± 3.16 ^a^ 6.89 (2.0–12.25)	7.15 ± 3.33 ^a^ 7.00 (2.25–13.50)	6.76 ± 3.30 ^a^ 6.63(1.50–12.0)
DFI, %	10.16 ± 7.97 ^a^ 7.86 (2.90–26.27)	15.15 ± 12.71 ^b^ 10.78 (2.54–41.72)	18.08 ± 11.97 ^b^ 13.75 (3.39–38.07)
LH, mIU/mL	3.24 ± 1.37 ^a^ 3.01 (1.42–5.67)	3.44 ± 1.46 ^a^ 3.31 (1.46–6.21)	3.56 ± 1.68 ^a^ 3.11 (1.53–7.30)
FSH, mIU/mL	3.55 ± 2.09 ^a^ 3.10 (1.26–7.18)	3.83 ± 2.51 **^ab^** 3.08 (1.39–8.58)	4.51 ± 3.61 ^b^ 3.75 (1.24–10.24)
Testosterone, nmol/L	24.32 ± 8.09 ^a^ 23.62 (12.93–37.97)	17.29 ± 6.53 ^b^ 15.57 (9.61–29.00)	14.24 ± 6.05 ^c^ 13.88 (7.33–25.92)
Estradiol, nmol/L	0.207 ± 0.074 ^a^ 0.196 (0.114–0.324)	0.204 ± 0.074 ^a^ 0.183 (0.125–0.364)	0.184 ± 0.052 ^b^ 0.173 (0.123–0.288)
Inhibin B, pg/mL	192.2 ± 66.9 ^a^ 184.2 (96.2–311.2)	182.3 ± 69.6 ^a^ 170.7 (86.7–311.8)	162.4 ± 64.3 ^b^ 165.5 (36.5–253.8)
Leptin, ng/mL	3.99 ± 3.90 ^a^ 2.75 (0.71–10.87)	10.66 ± 7.08 ^b^ 8.50 (3.98–29.11)	22.32 ± 11.95 ^c^ 21.07 (10.79–48.97)
TG, mmol/L	0.98 ± 0.58 ^a^ 0.85 (0.39–2.09)	1.58 ± 0.95 ^b^ 1.38 (0.54–3.61)	2.02 ± 1.12 ^c^ 1.71 (0.72–3.61)
TC, mmol/L	3.99 ± 0.90 ^a^ 3.91 (2.70–5.59)	4.60 ± 1.07 ^b^ 4.55 (2.88–6.23)	4.74 ± 1.02 ^b^ 4.63 (3.25–6.50)
HDL-C, mmol/L	1.25 ± 0.31 ^a^ 1.22 (0.83–1.77)	1.08 ± 0.35 ^b^ 1.05 (0.58–1.71)	1.08 ± 0.35 ^b^ 1.01 (0.67–1.82)
LDL-C, mmol/L	2.35 ± 0.94 ^a^ 2.27 (1.03–4.01)	3.08 ± 1.31 ^b^ 2.97 (1.18–5.44)	3.34 ± 1.30 ^b^ 3.11 (1.65–5.75)
Fasting glucose, mmol/L	4.6 ± 1.1 ^a^ 4.5 (3.4–5.9)	5.2 ± 0.8 ^b^ 5.1 (4.1–6.8)	5.9 ± 2.3 ^c^ 5.5 (4.2–7.6)
Uric acid, μmol/L	339 ± 74 ^a^ 331 (226–456)	406 ± 107 ^b^ 395 (271–588)	465 ± 161 ^c^ 422 (315–790)
Serum zinc level, µmol/L	21.0 ± 7.3 ^a^ 19.2 (13.9–35.3)	27.7 ± 13.0 ^b^ 23.8 (15.5–56.8)	34.5 ± 16.1 ^c^ 30.5 (16.9–69.2)
Seminal zinc content, µmol/ejaculate	6.03 ± 4.48 ^a^ 4.93 (1.17–14.70)	6.34 ± 5.07 ^a^ 5.26 (0.97–14.75)	4.83 ± 3.69 ^b^ 4.05 (0.71–12.56)

Note: The stratification of men into three groups was carried out by waist circumference in accordance with the WHO recommendations [12]. Values are presented as mean (SD) and median (5–95th percentile). BMI, body mass index; BTV, paired testicular volume; DFI, sperm DNA fragmentation index; LH, luteinizing hormone; FSH, follicle-stimulating hormone; TG, triglycerides; TC, total cholesterol; HDL-C, high-density lipoprotein cholesterol; LDL-C, low-density lipoprotein cholesterol; ^a, b, c^—comparisons with different superscripts within variable are significant (*p* < 0.05).

**Table 2:** Distribution of anthropometric, semen, hormonal, zinc, and metabolic parameters in participants by waist circumference associated with testosterone deficiency.

Variable	Non-Obese Control (WC < 94 cm)		Excess of Abdominal Fat (102 > WC ≥ 94 cm)		Abdominal Obesity (WC ≥ 102 cm)	
	Normal Testosterone, n = 1066	Testosterone Deficiency, n = 34	Normal Testosterone n = 89	Testosterone Deficiency n = 21	Normal Testosterone n = 45	Testosterone Deficiency n = 28
Age, years	23.9 ± 5.6 22.0 (18.0–33.0)	29.5 ± 11.1 * 25.5 (18.0–55.0)	30.4 ± 8.8 29.0 (20.0–47.0)	29.2 ± 7.0 28.0 (22.0–42.0)	32.0 ± 10.1 30.0 (20.0–52.0)	32.2 ± 8.2 32.0 (20.0–47.0)
Body weight, kg	74.7 ± 9.4 74.0 (60.0–90.3)	75.7 ± 7.7 78.0 (59.0–88.0)	93.5 ± 9.3 93.2 (79.0–106.9)	93.0 ± 6.5 93.0 (83.10–103.2)	106.8 ± 11.6 105.0 (90.0–127.0)	105.1 ± 10.3 105.0 (90.0–130.0)
Height, cm	179.0 ± 6.7 179.0 (169.0–190.0)	176.8 ± 6.0 176.0 (165.0–186.0)	181.5 ± 6.9 182.0 (169.0–193.0)	182.0 ± 7.6 183.5 (166.0–190.0)	183.1 ± 7.2 183.0 (173.0–193.0)	177.9 ± 5.2 * 178.5 (169.5–186.0)
BMI, kg/m^2^	23.3 ± 2.6 23.2 (19.1–27.7)	24.2 ± 2.5 24.4 (19.9–28.9)	28.4 ± 2.4 28.4 (25.1–32.6)	28.1 ± 1.8 27.8 (25.8–30.7)	31.9 ± 3.2 31.3 (27.8–38.0)	33.2 ± 2.9 33.8 (29.1–38.8)
Waist circumference, cm	80.3 ± 6.3 80.0 (70.0–91.0)	83.3 ± 6.5 * 84.0 (72.0–92.0)	97.0 ± 2.2 97.0 (94.0–101.0)	97.0 ± 2.7 97.0 (94.0–101.0)	107.0 ± 5.4 105.0 (102.0–118.0)	108.5 ± 5.4 106.5 (103.0–120.0)
Hip circumference, cm	96.1 ± 6.0 96.0 (87.0–106.0)	97.4 ± 5.0 96.5 (90.0–106.0)	106.6 ± 5.0 106.0 (99.0–114.0)	107.3 ± 4.5 107.0 (101.0–115.0)	113.8 ± 6.2 113.0 (105.0–126.0)	112.4 ± 4.7 111.0 (107.0–120.0)
BTV, mL	41.8 ± 8.3 40.0 (28.0–50.0)	41.9 ± 10.8 40.0 (22.0–60.0)	45.8 ± 7.7 50.0 (32.0–59.3)	44.6 ± 9.3 44.0 (30.0–60.0)	42.6 ± 10.2 40.0 (25.0–60.0)	42.6 ± 10.0 42.0 (30.0–60.0)
Sexual abstinence, days	4.8 ± 4.9 4.0 (2.0–8.0)	4.8 ± 5.3 4.0 (1.0–14.0)	5.1 ± 6.6 4.0 (2.0–7.0)	4.4 ± 3.0 4.0 (2.0–7.0)	3.9 ± 1.5 4.0 (2.0–6.0)	4.4 ± 3.2 3.0 (2.0–14.0)
Semen volume, mL	3.8 ± 1.7 3.6 (1.4–6.8)	3.8 ± 1.7 3.6 (1.3–7.1)	3.8 ± 1.8 3.5 (1.3–6.6)	3.3 ± 1.6 3.1 (1.3–8.4)	3.0 ± 1.5 3.0 (0.9–5.5)	2.9 ± 1.5 2.5 (1.1–5.4)
Total sperm count, ×10^6^/ejaculate	252.7 ± 223.8 206.1 (15.0–673.1)	238.4 ± 195.2 169.3 (30.6–754.3)	227.1 ± 211.7 172.1 (7.8–539.8)	162.0 ± 169.3 85.0 (4.4–596.4)	138.1 ± 112.1 110.8 (5.3–390.3)	144.5 ± 156.4 106.7 (0.0–413.1)
Sperm concentration, ×10^6^/mL	67.78 ± 52.38 55.39 (5.63–167.00)	64.86 ± 49.55 55.14 (9.00–161.16)	63.69 ± 51.72 50.25 (4.37–159.25)	52.38 ± 56.30 35.25 (4.41–184.44)	45.27 ± 33.51 35.24 (6.02–98.71)	47.64 ± 48.59 32.55 (0.00–153.00)
Progressive motility, %	49.0 ± 26.8 49.2 (4.6–90.9)	46.1 ± 25.2 47.3 (4.3–95.5)	46.3 ± 28.8 46.0 (3.1–91.5)	40.1 ± 31.5 31.6 (6.2–93.0)	34.7 ± 25.1 29.4 (1.9–82.4)	41.8 ± 31.3 41.3 (0.2–88.5)
Normal morphology, %	7.04 ± 3.13 6.75 (2.0–12.25)	7.27 ± 3.76 7.38 (1.50–14.75)	7.44 ± 3.34 7.25 (2.50–13.75)	5.94 ± 3.11 4.75 (2.0–10.82)	6.79 ± 3.12 6.75 (1.50–11.00)	6.70 ± 3.70 6.25 (2.00–14.00)
DFI, %	10.12 ± 8.14 7.85 (2.90–26.25)	11.86 ± 4.01 12.52 (3.55–16.95)	14.59 ± 13.02 9.52 (2.54–41.72)	17.49 ± 12.39 12.04 (5.19–32.08)	15.37 ± 11.23 13.41 (3.20–38.07)	25.46 ± 11.50 * 25.75 (10.65–40.79)
LH, mIU/mL	3.24 ± 1.34 3.01 (1.43–5.66)	3.27 ± 1.41 3.09 (1.42–5.47)	3.45 ± 1.43 3.30 (1.67–6.10)	3.38 ± 1.63 3.34 (1.18–6.21)	3.63 ± 1.61 3.16 (1.86–7.11)	3.44 ± 1.80 2.84 (1.38–7.57)
FSH, mIU/mL	3.52 ± 2.00 3.09 (1.27–7.12)	4.31 ± 3.60 3.77 (1.62–8.38)	3.83 ± 2.60 3.07 (1.31–8.58)	3.84 ± 2.16 3.33 (2.10–7.36)	3.94 ± 2.32 3.74 (1.24–8.18)	5.43 ± 4.97 * 3.83 (1.74–20.09)
Testosterone, nmol/L	24.63 ± 7.51 23.80 (14.08–37.95)	10.77 ± 1.79 * 11.30 (5.93–12.12)	18.95 ± 6.16 17.35 (12.68–29.34)	10.29 ± 1.44 * 10.58 (7.43–11.85)	17.61 ± 5.16 16.52 (12.84–25.95)	8.81 ± 2.17 * 8.69 (5.46–11.71)
Estradiol, nmol/L	0.207 ± 0.073 0.197 (0.114–0.322)	0.182 ± 0.061 0.182 (0.112–0.320)	0.207 ± 0.072 0.187 (0.128–0.337)	0.192 ± 0.084 0.161 (0.118–0.364)	0.195 ± 0.057 0.185 (0.111–0.312)	0.166 ± 0.034 0.156 (0.123–0.231)
Inhibin B, pg/mL	193.1 ± 67.1 184.7 (96.7–312.0)	173.7 ± 53.7 176.8 (70.6–270.0)	182.3 ± 68.4 177.7 (90.5–311.5)	182.2 ± 76.0 163.9 (72.0–311.8)	166.8 ± 53.0 171.0 (72.3–249.2)	155.4 ± 79.8 159.1 (2.3–253.8)
Leptin, ng/mL	4.02 ± 3.92 2.77 (0.74–11.30)	2.66 ± 2.02 1.75 (0.57–6.29)	10.65 ± 7.38 7.90 (3.96–30.11)	10.69 ± 6.20 8.81 (4.41–25.74)	20.54 ± 9.45 20.51 (8.89–48.97)	36.46 ± 17.05 * 31.01 (22.80–55.56)
TG, mmol/L	0.97 ± 0.57 0.84 (0.38–2.07)	1.19 ± 0.73 1.06 (0.25–2.88)	1.55 ± 0.95 1.35 (0.56–3.56)	1.71 ± 0.96 1.66 (0.35–3.71)	1.86 ± 0.96 1.56 (0.66–3.54)	2.28 ± 1.31 * 1.99 (0.77–3.66)
TC, mmol/L	4.00 ± 0.90 3.92 (2.71–5.59)	3.95 ± 0.89 3.85 (2.95–5.68)	4.61 ± 1.02 4.55 (2.89–6.23)	4.58 ± 1.26 4.51 (2.66–6.58)	4.56 ± 0.80 4.56 (3.10–5.71)	5.02 ± 1.26 * 4.75 (3.36–7.30)
HDL-C, mmol/L	1.26 ± 0.31 1.22 (0.85–1.77)	1.21 ± 0.31 1.17 (0.67–1.71)	1.08 ± 0.33 1.05 (0.59–1.69)	1.09 ± 0.44 1.07 (0.58–1.87)	1.12 ± 0.37 1.06 (0.68–1.82)	1.01 ± 0.31 0.95 (0.67–1.57)
LDL-C, mmol/L	2.35 ± 0.93 2.27 (1.03–4.01)	2.52 ± 1.17 2.38 (1.04–3.96)	3.05 ± 1.24 2.93 (1.21–5.37)	3.22 ± 1.60 3.11 (1.13–6.20)	3.05 ± 1.20 2.94 (1.65–5.52)	3.80 ± 1.32 * 3.57 (1.65–5.75)
Fasting glucose, mmol/L	4.6 ± 1.1 4.5 (3.4–5.9)	4.8 ± 0.9 4.7 (3.2–6.0)	5.1 ± 0.8 5.0 (4.1–6.6)	5.5 ± 1.1 5.2 (4.1–6.9)	5.4 ± 1.0 5.1 (4.1–7.2)	6.6 ± 3.3 * 5.9 (4.7–7.7)
Uric acid, μmol/L	338 ± 73 331 (229–453)	372 ± 96 364 (213–599)	400 ± 98 388 (271–563)	435 ± 137 411 (286–596)	413 ± 115 383 (315–576)	546 ± 189 * 514 (363–942)
Serum zinc concentration, µmol/L	20.8 ± 7.1 19.2 (13.9–34.6)	26.2 ± 9.8 * 23.9 (13.7–45.2)	26.8 ± 12.3 23.8 (15.2–54.6)	31.5 ± 15.7 * 27.5 (16.0–71.6)	31.8 ± 15.4 26.0 (16.9–62.6)	39.0 ± 16.6 * 33.7 (19.4–69.2)
Seminal zinc content, µmol/ejaculate	6.05 ± 4.50 4.97 (1.17–14.75)	5.52 ± 3.71 4.36 (1.10–12.03)	6.69 ± 5.21 5.65 (1.22–16.29)	4.67 ± 4.08 2.97 (0.74–12.44)	4.80 ± 3.43 4.24 (0.94–12.56)	4.87 ± 4.15 3.92 (0.49–13.89)

Note: Values are presented as mean (SD) and median (5–95th percentile). BMI, body mass index; BTV, paired testicular volume; DFI, sperm DNA fragmentation index; LH, luteinizing hormone; FSH, follicle-stimulating hormone; TG, triglycerides; TC, total cholesterol; HDL-C, high-density lipoprotein cholesterol; LDL-C, low-density lipoprotein cholesterol; *—comparisons between groups with normal testosterone levels and those with TD are significant (*p* < 0.05).

**Table 3:** Anthropometric, semen, hormonal, zinc and metabolic indicators in participants with different levels of abdominal fat accumulation associated with a sedentary or physically active lifestyle.

Variable	Non-Obese Control (WC < 94 cm)		Abdominal Fat Accumulation (WC ≥ 94 cm)	
	Sedentary (n = 464)	Physical Activity (n = 308)	Sedentary (n = 86)	Physical Activity (n = 20)
Age, years	24.9 ± 6.1 * 23.0 (19.0–35.0)	22.5 ± 5.4 21.0 (18.0–31.0)	31.9 ± 9.9 29.0 (21.0–51.0)	30.8 ± 10.5 28.0 (18.5–51.5)
Body weight, kg	75.4 ± 9.5 74.7 (61.4–92.0)	78.8 ± 9.3 75.0 (61.9–92.0)	99.7 ± 12.9 * 97.7 (83.1–120.0)	96.1 ± 8.7 95.0 (80.0–112.0)
Height, cm	179.1 ± 6.6 179.0 (169.0–190.0)	179.2 ± 6.7 179.0 (168.0–191.0)	180.5 ± 7.0 181.0 (168.5–193.0)	182.1 ± 5.4 182.3 (172.0–189.5)
BMI, kg/m^2^	23.5 ± 2.7 23.4 (19.1–28.4)	23.6 ± 2.3 23.5 (20.0–27.2)	30.6 ± 3.3 * 30.1 (26.3–37.8)	29.0 ± 2.8 29.0 (25.2–34.6)
Waist circumference, cm	80.7 ± 6.5 81.0 (70.0–91.0)	80.0 ± 5.9 80.0 (71.0–90.0)	101.1 ± 5.8 * 100.0 (94.0–113.0)	98.2 ± 4.5 97.0 (94.0–107.5)
Hip circumference, cm	96.7 ± 6.0 97.0 (87.0–106.0)	96.5 ± 5.9 96.0 (88.0–106.0)	109.7 ± 5.8 110.0 (102.0–120.0)	108.3 ± 5.3 108.0 (101.0–119.0)
BTV, mL	42.7 ± 8.9 42.0 (27.0–54.0)	43.1 ± 8.2 45.0 (28.0–50.0)	45.2 ± 9.1 50.0 (30.0–60.0)	47.5 ± 8.9 50.0 (34.7–65.0)
Sexual abstinence, days	4.5 ± 4.1 4.0 (2.0–8.0)	5.1 ± 8.3 4.0 (2.0–14.0)	4.2 ± 2.6 4.0 (2.0–7.0)	3.8 ± 1.7 3.0 (2.0–7.0)
Semen volume, mL	3.9 ± 1.7 3.6 (1.4–7.1)	3.6 ± 1.7 3.4 (1.3–6.5)	3.3 ± 1.9 * 2.9 (1.0–6.7)	4.0 ± 2.0 3.7 (1.1–8.1)
Total sperm count, ×10^6^/ejaculate	262.6 ± 220.3 221.6 (13.9–660.8)	266.1 ± 234.2 205.8 (13.4–763.5)	202.0 ± 216.0 136.7 (5.8–539.4)	219.1 ± 172.8 192.1 (14.2–554.9)
Sperm concentration, ×10^6^/mL	68.49 ± 50.66 58.63 (6.25–166.26)	75.40 ± 61.20 54.75 (3.88–192.00)	59.96 ± 52.00 44.19 (4.41–159.25)	56.17 ± 41.40 46.90 (4.91–140.63)
Progressive motility, %	50.6 ± 26.9 51.8 (4.3–93.0)	51.5 ± 26.7 53.3 (4.8–92.4)	44.4 ± 29.4 41.6 (3.5–93.0)	38.7 ± 22.4 37.7 (6.8–82.1)
Normal morphology, %	6.89 ± 3.12 6.76 (1.75–11.75)	7.21 ± 3.12 7.00 (2.50–13.50)	6.96 ± 3.47 6.75 (1.75–13.50)	6.50 ± 2.87 7.13 (2.09–10.25)
DFI, %	9.89 ± 7.33 7.82 (3.35–24.39)	8.36 ± 5.65 6.99 (2.51–17.70)	11.04 ± 9.67 6.91 (2.54–32.08)	8.38 ± 0.42 8.44 (7.89–8.74)
LH, mIU/mL	2.93 ± 1.23 2.75 (1.30–5.30)	3.12 ± 1.25 2.92 (1.45–5.51)	3.12 ± 1.47 * 2.88 (1.37–6.10)	3.76 ± 1.93 3.22 (0.92–7.55)
FSH, mIU/mL	3.40 ± 2.38 2.90 (1.25–6.72)	3.74 ± 2.38 3.22 (1.34–7.23)	3.72 ± 2.75 3.08 (1.24–7.39)	4.66 ± 3.69 4.00 (0.79–12.72)
Testosterone, nmol/L	23.98 ± 8.27 22.84 (12.47–38.44)	26.32 ± 7.87 25.82 (15.64–38.15)	15.43 ± 5.86 * 14.24 (8.22–25.14)	22.58 ± 9.42 20.69 (12.23–39.02)
Estradiol, nmol/L	0.205 ± 0.083 0.193 (0.109–0.325)	0.205 ± 0.061 0.200 (0.108–0.314)	0.198 ± 0.075 0.177 (0.116–0.364)	0.202 ± 0.055 0.189 (0.137–0.309)
Inhibin B, pg/mL	201.0 ± 64.7 194.5 (102.2–320.7)	191.5 ± 69.0 186.0 (96.6–314.6)	185.4 ± 64.9 182.5 (72.3–300.3)	187.6 ± 58.5 194.7 (95.2–299.6)
Leptin, ng/mL	4.28 ± 3.61 3.26 (0.75–11.56)	3.53 ± 4.26 2.30 (0.63–9.85)	15.39 ± 10.90 * 11.38 (4.14–31.35)	10.54 ± 7.28 7.99 (1.70–25.84)
TG, mmol/L	1.03 ± 0.64 0.86 (0.40–2.22)	0.90 ± 0.52 0.77 (0.35–1.86)	1.75 ± 0.94 * 1.56 (0.67–3.66)	1.27 ± 0.75 1.00 (0.48–2.87)
TC, mmol/L	4.14 ± 0.92 4.07 (2.74–5.76)	4.02 ± 0.90 3.95 (2.77–5.52)	4.76 ± 1.01 * 4.69 (3.10–6.58)	4.25 ± 0.69 4.28 (3.17–5.57)
HDL-C, mmol/L	1.26 ± 0.28 1.23 (0.88–1.75)	1.28 ± 0.28 1.25 (0.89–1.77)	1.12 ± 0.32 1.09 (0.68–1.69)	1.16 ± 0.36 1.11 (0.66–1.86)
LDL-C, mmol/L	2.52 ± 1.00 2.42 (1.08–4.16)	2.39 ± 0.87 2.31 (1.15–3.95)	3.54 ± 1.36* 3.26 (1.90–6.10)	3.11 ± 1.12 2.92 (1.75–5.28)
Fasting glucose, mmol/L	4.6 ± 1.4 4.5 (3.3–5.9)	4.4 ± 0.7 4.3 (3.3–5.5)	5.3 ± 0.9 * 5.1 (4.2–7.2)	4.9 ± 0.8 5.0 (3.6–6.6)
Uric acid, μmol/L	339 ± 74 329 (240–452)	337 ± 68 328 (245–443)	415 ± 108 * 406 (288–564)	354 ± 79 350 (221–500)
Serum zinc level, µmol/L	20.6 ± 7.1 18.9 (13.7–35.1)	19.3 ± 6.7 17.9 (13.7–29.6)	28.7 ± 13.9 * 22.4 (15.5–60.2)	24.0 ± 10.9 21.6 (12.9–49.0)
Seminal zinc content, µmol/ejaculate	6.20 ± 4.55 4.97 (1.16–15.08)	5.93 ± 4.02 5.16 (1.20–13.44)	5.55 ± 4.74 4.35 (0.90–15.49)	6.50 ± 5.67 5.07 (1.41–18.66)

Note: Values are presented as mean (SD) and median (5–95th percentile). BMI, body mass index; BTV, paired testicular volume; DFI, sperm DNA fragmentation index; LH, luteinizing hormone; FSH, follicle-stimulating hormone; TG, triglycerides; TC, total cholesterol; HDL-C, high-density lipoprotein cholesterol; LDL-C, low-density lipoprotein cholesterol; *—comparisons between the sedentary and physical activity groups are significant (*p* < 0.05).

## Data Availability

Restrictions apply to the availability of some data of this study to preserve patient confidentiality. The corresponding author will on request detail the restrictions and other conditions under which access to some data may be provided.

## Abbreviations

The following abbreviations are used in this manuscript:

AO	Abdominal obesity
ROS	Reactive oxygen species
DFI	Sperm DNA fragmentation index
BMI	Body mass index
BTV	Bitesticular volume
WC	Waist circumference
LH	Luteinizing hormone
FSH	Follicle-stimulating hormone
TD	Testosterone deficiency
TG	Triglycerides
TC	Total cholesterol
HDL-C	High-density lipoprotein cholesterol
LDL-C	Low-density lipoprotein cholesterol
PA	Physical activity
SB	Sedentary behavior
